# Removal of Lead from Wastewater Using Synthesized Polyethyleneimine-Grafted Graphene Oxide

**DOI:** 10.3390/nano13061078

**Published:** 2023-03-16

**Authors:** Mohammed Al-Yaari, Tawfik A. Saleh

**Affiliations:** 1 Chemical Engineering Department, King Faisal University, P.O. Box 380, Al-Ahsa 31982, Saudi Arabia; 2 Chemistry Department, King Fahd University of Petroleum and Minerals, Dhahran 31261, Saudi Arabia

**Keywords:** lead removal, polyethyleneimine-grafted graphene oxide, polyethyleneimine, graphene oxide, adsorption, water purification

## Abstract

In this work, polyethyleneimine-grafted graphene oxide (PEI/GO) is synthesized using graphene, polyethyleneimine, and trimesoyl chloride. Both graphene oxide and PEI/GO are characterized by a Fourier-transform infrared (FTIR) spectrometer, a scanning electron microscope (SEM), and energy-dispersive X-ray (EDX) spectroscopy. Characterization results confirm that polyethyleneimine is uniformly grafted on the graphene oxide nanosheets and, thus, also confirm the successful synthesis of PEI/GO. PEI/GO adsorbent is then evaluated for the removal of lead (Pb^2+^) from aqueous solutions, and the optimum adsorption is attained at pH 6, contact time of 120 min, and PEI/GO dose of 0.1 g. While chemosorption is dominating at low Pb^2+^ concentrations, physisorption is dominating at high concentrations and the adsorption rate is controlled by the boundary-layer diffusion step. In addition, the isotherm study confirms the strong interaction between Pb^2+^ ions and PEI/GO and reveals that the adsorption process obeys well the Freundlich isotherm model (R^2^ = 0.9932) and the maximum adsorption capacity (q_m_) is 64.94 mg/g, which is quite high compared to some of the reported adsorbents. Furthermore, the thermodynamic study confirms the spontaneity (negative ΔG° and positive ΔS°) and the endothermic nature (ΔH° = 19.73 kJ/mol) of the adsorption process. The prepared adsorbent (PEI/GO) offers a potential promise for wastewater treatment because of its fast and high uptake removal capacity and could be used as an effective adsorbent for the removal of Pb^2+^-ions and other heavy metals from industrial wastewater.

## 1. Introduction

Due to the rapid expansion of industrial development and population growth, there are now significantly higher levels of water contaminants. Due to their disposal and thus, potential accumulation in soil and water, as well as along the production food chains, the highly detrimental effects of toxic metal ions among the various water pollutants have been confirmed. These pollutants include lead ions (Pb^2+^) that are toxic and non-biodegradable [1].

The primary sources of metal ions are industrial processes, particularly those that are related to batteries, mining, and many other parts. These processes also produce significant amounts of wastewater that is metal-ion polluted. The challenges for metal-ion pollution and the chance for the development of different metal-ion elimination methods are the rules forced on the removal of metal-ion waste from wastewater and the struggle in accomplishing their acceptable level of elimination. Thus, there is a financial and environmental need to efficiently treat wastewater containing toxic heavy metals before releasing them into the environment. Generally, precipitation, ion exchange, adsorption, filtration/reverse osmosis, coagulation-flocculation, and the oxidative process are some of the conventional physical and chemical methods used to treat industrial wastewater [2,3,4,5]. In addition, the pulsed laser technique was recently used for the dechlorination of Cl-based pollutants [6] and the production of nanocomposites that can be used for the detection of nitro-organic compounds [7]. The benefits and drawbacks of some of the conventional techniques used to remove water pollutants are presented and logically debated in a few review publications [2,3,4,5] and summarized in Table 1. 

Adsorption is a surface process, and to enhance the whole exposed surface area and facilitate faster fluid passage, adsorbents often have porous architectures. Due to its cost-effectiveness, environmental friendliness/easiness, the regeneration potential of the materials for many reuses, and better removal efficiency, the adsorption process is highly favored over other techniques in the removal of heavy metals and dyes from wastewater.

There are several adsorbents used for Pb^2+^ removal from aqueous solutions including agricultural waste-based biosorbents such as Albizia lebbeck pods [8], cassava peels [9], brewed tea waste [10], sugarcane bagasse [11], mango and jamun seed covers with kernels [12], and Parkia speciosa pods [13]. 

However, composites are alternative promising materials for metal adsorption. For instance, a composite of polymer carbon nanofiber was reported for mercury (Hg) removal from aqueous media [14]. Another novel composite of polyacrylate-modified carbons was reported for Hg removal from water [15]. Graphene with two-dimensional carbon structures has high chemical stability and great surface area [16]. Thus, it is a promising candidate for various uses including the treatment of wastewater. Graphene oxide (GO) possesses oxygenated functional groups including the –O–, –OH, and –COOH groups. Such functionality and active sites make the GO an effective adsorbing material for inorganic and organic pollutants. Composites of GO with other components can be a good way to further improve GO efficiency toward the removal of pollutants. For example, composites of graphene oxide and chitosan were reported for the elimination of toxic metal ions and some organic pollutants from water [17]. Composites of functionalized GO with chitosan were also reported for lead-ion removal from water [18]. Ethylene diamine tetra-acetic acid-modified GO was also reported for metal-ion removal including lead ions. GO has several functionalities and active sites, and ethylene diamine tetra-acetic acid has a chelating agent for metal ions [19]. A combination of GO with chitosan and ethylene diamine tetra-acetic acid was also reported to have a good ability for metal-ion removal [1].

Polyamide-amine-based polymers present molecular structures with developed nanocavities and several terminal amino groups that enhance the hydrophilicity and the interactions with toxic metal ions [20]. Materials with nitrogen-chelating ligands, like amino groups, are highly effective adsorbents in the adsorption of toxic heavy metal ions [21]. Polyamide functionalized graphene oxide has been reported as an adsorbent for pollutants removal from water, such as cadmium, arsenite, ciprofloxacin, tetracycline, and ivermectin [22]. In addition, polyacrylamide-grafted magnetic reduced graphene oxide nanocomposite was used as an adsorbent for the removal of Congo red (an anionic dye) from aqueous solutions [23]. Zhao and Liu (2014) synthesized poly (tert-butyl acrylate)-grafted graphene oxide adsorbent to be used for the removal of tetra-bromo-bisphenol A from aqueous solution, and an adsorption capacity of 22.2 mg/g at pH 7 was reported [24].

Based on the literature, in this study, a novel and efficient adsorbent was designed to combine the great properties of graphene oxide and polyamide. Thus, the graphene oxide was grafted with polyethyleneimine to form an efficient adsorbent for the adsorption of highly toxic lead metal ions. The design of the composite in this work was based on the principle of introducing more active sites on the surface of graphene oxide to further enhance its affinity to capture toxic metal ions. The principle was to enhance the contact area of the adsorbent, thus further improving the pollutants’ removal. The adsorption parameters were all comprehensively investigated, and the related kinetics and isotherm models were employed to evaluate the process. Furthermore, thermodynamic investigation at different temperatures was performed as well.

## 2. Materials and Methods

### 2.1. Reagents 

The nitric acid, polyethyleneimine, trimesoyl chloride, and hexane reagents used in the synthesis of the polyethyleneimine-grafted graphene oxide (PEI/GO) adsorbent were purchased from Sigma Aldrich Company (St. Louis, MO, USA). 

A 1000 mg/L stock solution of lead was prepared by dissolving lead nitrate in deionized distilled water. A mixture of pollutants was also prepared by dissolving the pollutants in deionized distilled water to prepare 200 ppm of each pollutant. The graphene oxide was prepared using Hummer’s method.

### 2.2. Synthesis and Characterization

For the synthesis of PEI/GO, the following steps were conducted. Initially, 0.1 M nitric acid was added to graphene and refluxed for 3 h at 90 °C to form oxygen-containing groups on its surface. The system was then given time to reach room temperature. The obtained graphene with oxygen-containing groups, such as hydroxyl groups, was then filtered and allowed to dry at 110 °C. 

**Figure 1:**
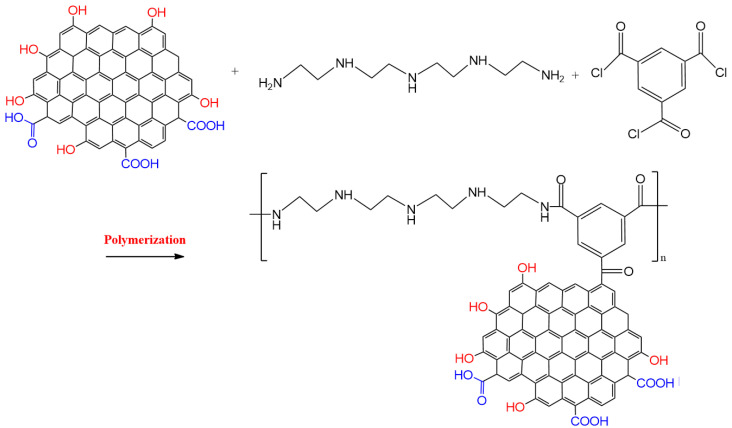
Schematic illustration of the formation of the polyethyleneimine-grafted graphene oxide (PEI/GO).

Interfacial polymerization was used to graft polymer chains onto graphene oxide. The obtained graphene oxide was mixed and dispersed with 2% *w*/*v* of polyethyleneimine in water, by sonication. After 3 h, trimesoyl chloride (0.10% *w*/*v*) in hexane, was dropwise introduced into the system under vigorous stirring. To attain complete polymerization, the system was stirred for one day. PEI/GO was separated and rinsed with solvent. The obtained PEI/GO was dried in a freeze-dryer. Figure 1 illustrates the formation of the synthesized PEI/GO. 

A Nicolet 6700 Fourier-transform infrared (FTIR) spectrometer, manufactured by Thermo-Fisher Scientific Co., Carlsbad, CA, USA, was used to determine the adsorbent (graphene oxide and PEI/GO) functional groups using the pellet formation method. In addition, a field-emission scanning electron microscope (SEM) equipped with energy-dispersive X-ray (EDX) spectroscopy, manufactured by TESCAN, Kohoutovice, Czech Republic, was used for the characterization of the graphene oxide and PEI/GO surface morphology and elemental analysis.

### 2.3. Batch Adsorption Procedure

Solutions with various concentrations of lead ions (Pb^2+^) were prepared by dilution from the stock solution using distilled water. NaOH and HCl solutions of 0.1 M were used to control the solution acidity (pH). All batch experiments were performed to remove Pb^2+^ from 20 mL of aqueous solutions. Initially, the optimum values of the adsorption parameters, including pH, contact time, adsorbate initial concentration, and adsorbent dosage, were determined. Hence, pH values ranging between 3 and 7, Pb^2+^ initial concentrations ranging between 50 and 400 mg/L, and 0.005 to 0.2 g of PEI/GO dosages were tested. The optimum values resulting in the highest Pb^2+^ removal % were used for the kinetic, isotherm, and thermodynamic investigations. Adsorbent was added to the Pb^2+^ solution in a centrifugal tube and shaken at 150 rpm using a shaker (Selecta Multimatic-55; Barcelona, Spain) till reaching equilibrium at different temperatures (23, 38, 53, and 68 °C). The sampling was performed at predetermined times (every 10 min). Then, the adsorbent was filtered, and the aliquots were analyzed by inductively coupled plasma (ICP) to monitor the Pb^2+^ removal efficiency. To ensure accuracy and results reproducibility, all adsorption experiments were carried out in triplicate, and the average values of the measured concentrations were considered in the calculations. In addition, the values of the standard deviation were reported. 

The Pb^2+^ removal (%) was calculated as:

(1) $$\mathrm{removal}\;\left(\%\right)=\frac{\left(C_{i}-{\;C}_{f}\right)}{C_{i}}\times 100$$

where C_i_ (mg/L) and C_f_ (mg/L) are the initial and final Pb^2+^ concentrations, respectively.

## 3. Results and Discussion

### 3.1. Morphological and Chemical Characterization

**Figure 2:**
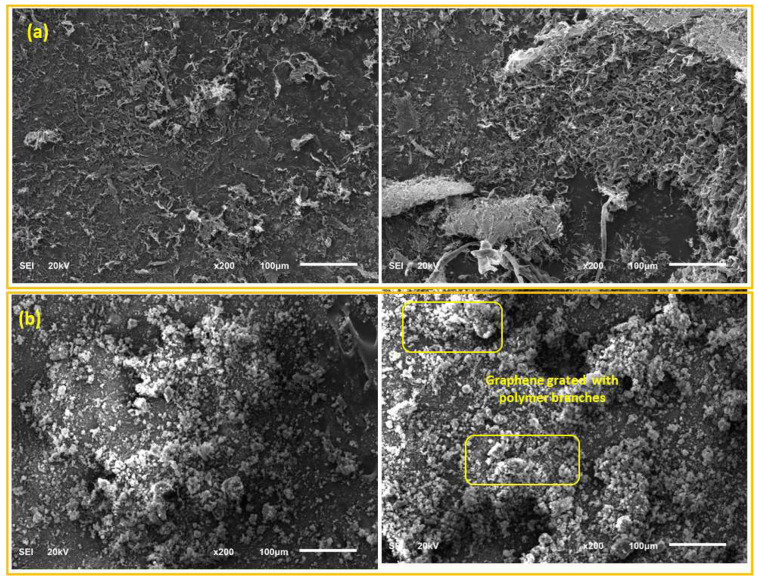
Scanning electron microscopy (SEM) images of (**a**) graphene oxide and (**b**) PEI/GO.

The morphology, structure, and surface state of graphene oxide and PEI/GO were evaluated using SEM analysis. Figure 2a,b displays the representative SEM images of the graphene oxide and PEI/GO, respectively. The SEM images indicate that the graphene oxide was of nanosheet shape with a smooth surface compared to PEI/GO. The SEM images of PEI/GO indicate the formation of polymer branches on the graphene oxide demonstrating that the surfaces of the prepared PEI/GO are quite different from the pure graphene oxide. 

**Figure 3:**
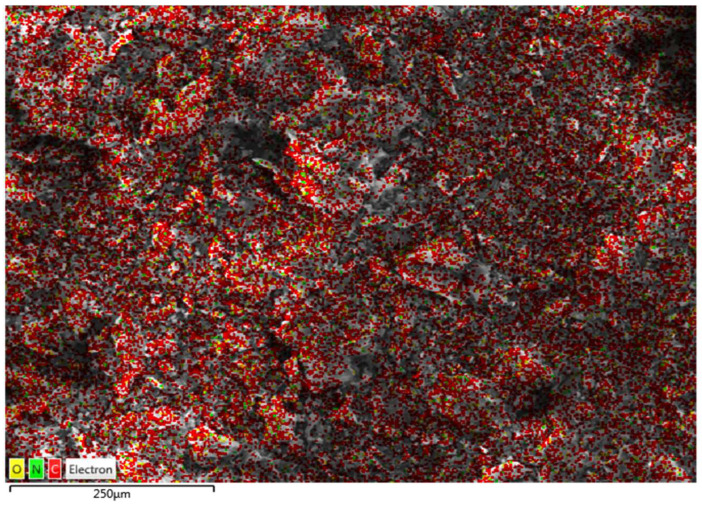
Mapping image indicating a uniform distribution of the N (from polyethyleneimine) among O and C in PEI/GO.

**Figure 4:**
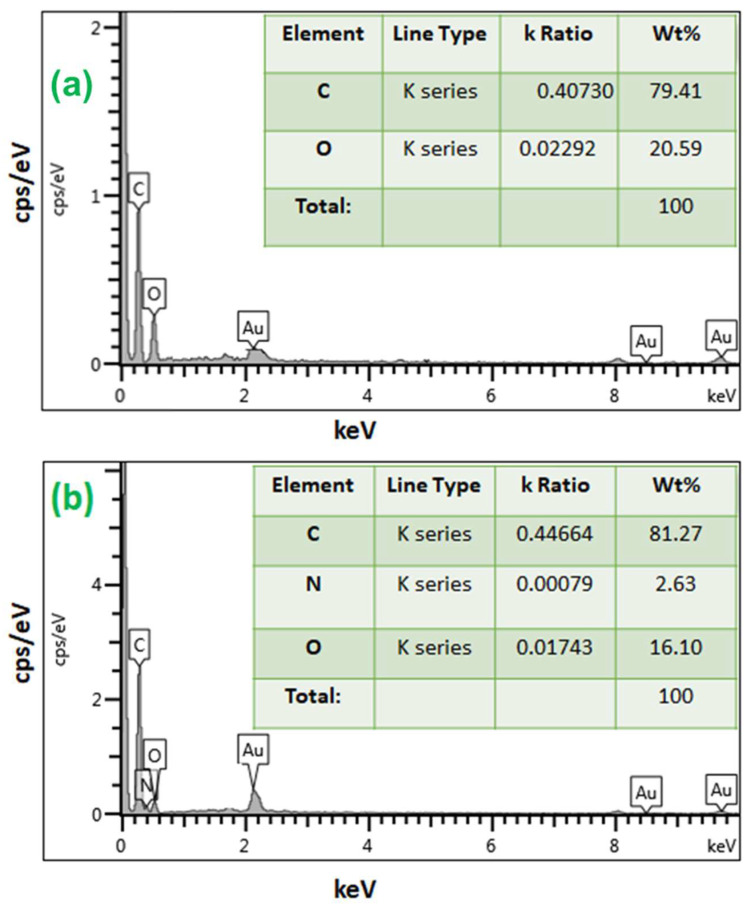
Energy-dispersive X-ray (EDX) spectra of (**a**) graphene oxide and (**b**) PEI/GO.

Figure 3 displays the mapping image, indicating a uniform distribution of the N (from polyethyleneimine) among O and C in PEI/GO. This indicates that the polymer of polyethyleneimine and trimesoyl chloride was uniformly grafted on the graphene oxide nanosheets. The compositional information of the EDX spectrum in Figure 4a exhibits the presence of C and O on graphene oxide, while Figure 4b exhibits the presence of C, O, and N elements on the PEI/GO adsorbent. The detected N signal provides powerful evidence for the successful grafting of graphene oxide with polymer chains. The tables (inset in Figure 4a,b) provide EDX analysis of each element of the prepared PEI/GO adsorbent.

**Figure 5:**
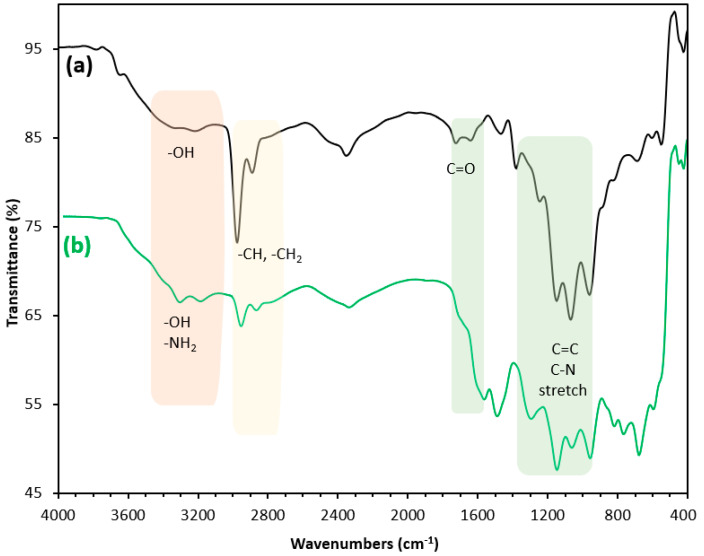
Fourier-transform infrared (FTIR) spectra of (a) graphene oxide and (b) PEI/GO.

To confirm the successful synthesis of PEI/GO, the structure of the materials was analyzed by FTIR. As shown in Figure 5, a broad and intense band in the region around 3000 to 3600 cm^−1^ is assigned for stretching vibrations of the hydroxyl group. The bands at 1726 cm^−1^, 1426 cm^−1^, 1375 cm^−1^, 1226 cm^−1^, and 1050 cm^−1^ can be attributed to C=O stretching vibration, deformation of O–H, vibration of C–OH, vibration of C–O (epoxy), and vibration of C–O (alkoxy), respectively [25]. 

The characteristic bands at 2935 cm^−1^ and 2835 cm^−1^ were assigned to the bending vibration of the alkyl group in the polymer chains [26]. The intense adsorption at 700 cm^−1^ can be ascribed to the benzene ring. The two stretching bands at 3400 cm^−1^ and 3280 cm^−1^ were ascribed to –NH_2_ or –NH [27,28]. The intense single peak at 1630 cm^−1^ was assigned to the bending vibration of N–H in the plane [29]. The appearance of –NH–CO– bands at 1650 cm^−1^ and 1545 cm^−1^ in the spectrum of PEI/GO confirmed that the acyl chloride group was successfully bound to the amino group on polyethyleneimine. These characterization results verified that the PEI/GO had been successfully prepared.

### 3.2. Adsorption Study

#### 3.2.1. Adsorption Performance of Graphene Oxide and PEI/GO

**Figure 6:**
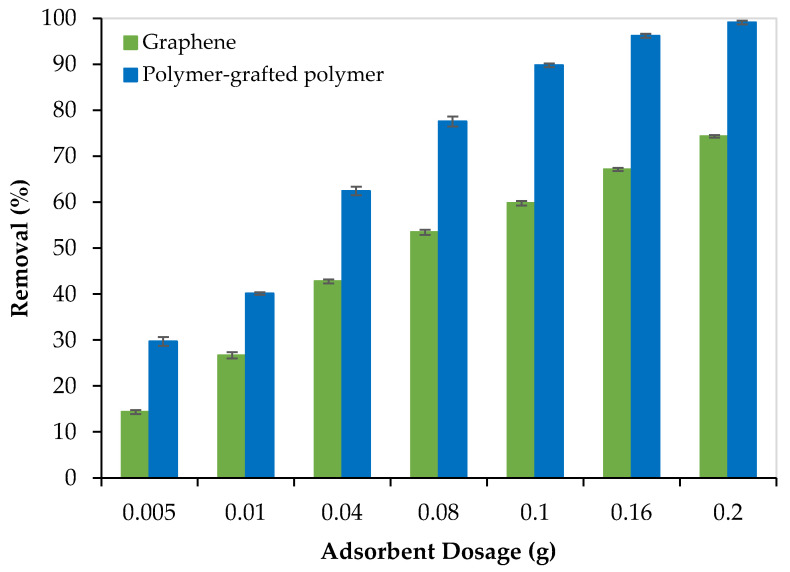
The removal performance of Pb^2+^ from aqueous solutions by graphene oxide and PEI/GO (pH = 6, Pb^2+^ concentration = 300 ppm, and 23 °C).

Both adsorbents (graphene oxide and PEI/GO) were evaluated for the removal of Pb^2+^ from aqueous solutions. For this purpose, the adsorption capacity of each adsorbent was evaluated for the removal of Pb^2+^ from 300 ppm water solutions using different dosages of adsorbent ranging from 0.005 to 0.2 g. Each experiment lasted for 120 min and was repeated three times to ensure the reproducibility of the results. Results are presented in Figure 6.

As the adsorbent dosage increased, the adsorption capacity (Pb^2+^ removal %) increased, and this can be attributed to the increase in the number of active sites. In addition, as presented in Figure 6, PEI/GO showed a better removal performance compared to graphene oxide and this can be credited to the formation of polymeric branches on the graphene oxide surface as illustrated by the SEM images (Figure 2b) and confirmed by the image mapping (Figure 3), EXD (Figure 4b), and FTIR (Figure 5). These formed polymeric chains offered extra adsorption active sites and more affinity to adsorb Pb^2+^ ions (more details are described in Section 3.5).

#### 3.2.2. Adsorption Optimum Parameters

The adsorption parameters including pH, Pb^2+^ initial concentrations, PEI/GO dosage, and contact time were initially optimized. The optimum values result in the highest removal %.

**Effect of pH**

**Figure 7:**
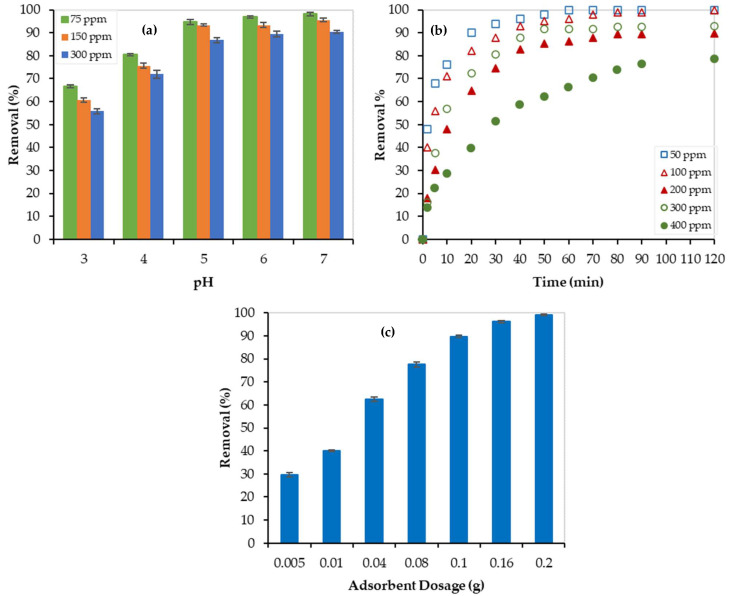
Removal of Pb^2+^ by PEI/GO at 23 °C: (**a**) effect of pH, (**b**) effect of Pb^2+^ initial concentration and contact time, and (**c**) effect of adsorbent dosage.

The effect of solution acidity (pH) on the Pb^2+^ removal by PEI/GO was investigated at 23 °C and 1 atm. Different water solutions with Pb^2+^ initial concentrations of 75 ppm, 150 ppm, and 300 ppm were treated with 0.1 g PEI/GO at different pH values, and results are presented in Figure 7a. At low pH (acidic solution), the removal % was low and this can be attributed to the protonation of the carbonyl groups and some of the PEI/GO active sites were occupied by the positive H^+^ rather than the Pb^2+^ positive ions. However, the removal % of Pb^2+^ increased as pH increased (i.e., as H^+^ ion concentrations decreased). At pH = 6, the removal % of Pb^2+^ reached the maximum at 97%, 94%, and 90% for the solutions of 75, 150, and 300 ppm of Pb^2+^ ions, respectively. Further increase in the pH value (pH = 7) has no significant increase in the Pb^2+^ removal %, which can be due to a balance between the reduction in H^+^ ions and the formation of the OH^−^ ions. While the H^+^ acts as a competitor for Pb^+2^, the OH^−^ acts as a competitor for NO_3_^2−^. With a further increase in pH (>7), the removal % is expected to decrease because of the concentration of the OH^−^ ions and the formation of some water-soluble lead complexes. Therefore, the pH value of 6 was maintained during the next batch of experiments.

**Effect of Contact Time**

The adsorption efficiency’s dependency on contact time is illustrated in Figure 7b. As shown in this figure, there were two stages of removal of Pb^2+^ by PEI/GO. The first one (sharp increase) showed a rapid adsorption process, and the second stage showed a stable adsorption process of Pb^2+^ (plateau curve), which implies reaching equilibrium. Generally, a fast Pb^2+^ removal by PEI/GO was noticed. For instance, after 10 min the Pb^2+^ removal % reached 76%, 71%, 57%, 48%, and 29% for the 50, 100, 200, 300, and 400 ppm Pb^2+^ aqueous solutions, respectively. This promising finding can be attributed to the sufficient adsorption-active sites offered by the polymeric branches on the graphene oxide surface, as discussed earlier, allowing the rapid removal of Pb^2+^ in the first stage. After that, the adsorption-active sites became occupied and thus equilibrium was reached. The maximum removal % of 100%, 100%, 93%, 90%, and 79% were reached for the 50, 100, 200, 300, and 400 ppm solutions, respectively. Since all experiments reached equilibrium within 120 min, batch experiments lasted for 120 min.

**Effect of Adsorbate (Pb^2+^) Initial Concentrations**

The high level of Pb^2+^ concentration in industrial wastewater ranges between 200 and 500 ppm [30]. Therefore, 0.1 g of PEI/GO was used to adsorb Pb^2+^ from aqueous solutions of 50, 100, 200, 300, and 400 ppm Pb^2+^ concentrations at ambient conditions (23 °C and 1 atm). As shown in Figure 7b, as the initial concentration of Pb^2+^ increased, the removal % decreased, and this can be linked to the ions’ competition for the available number of active sites on the adsorbent surface (i.e., the ions’ occupation of PEI/GO-active sites). A further increase in Pb^2+^ initial concentration produces more Pb^2+^ ions till reaching equilibrium (no more sites are available for adsorption). At the end of each experiment (after 120 min), Pb^2+^ removal % of 100%, 100%, 93%, 90%, and 79% were reached for the 50, 100, 200, 300, and 400 ppm solutions, respectively. This promising finding can lead to the potential use of PEI/GO to treat wastewater with high Pb^2+^ concentrations. In addition, all treated solutions reached equilibrium within 120 min.

**Effect of Adsorbent (PEI/GO) Dosage**

For this purpose, 20 mL water solutions, with a Pb^2+^ concentration of 300 ppm, were treated with different PEI/GO dosages ranging between 0.005 and 0.2 g. As the adsorbent dosage increased from 0.005 g to 0.1 g, the adsorbate removal % increased from 30% to 90%, as shown in Figure 7c. Then, a further increase in the adsorbent mass resulted in no substantial increase in the adsorption removal %. This trend can be credited to the active sites’ adequacy for the available lead ions. At low PEI/GO dosage, the active sites were insufficient, and when the dosage increased, the removal efficiency increased. Higher adsorbent dosage offers more active sites for a fixed adsorbate concentration (300 ppm) leading to a constant removal %. Twenty milligrams of PEI/GO were enough for the 100% removal of Pb^2+^ from water solutions. However, the use of 0.1 g of PEI/GO resulted in 90% adsorption removal, and thus, it was economically recommended for the kinetic and isotherm studies. 

### 3.3. Kinetic and Isotherm Studies

#### 3.3.1. Kinetic Study

The kinetic investigation aims to obtain the appropriate adsorption mechanism and to determine the limiting step of the adsorption rate. For this purpose, the equations of pseudo-first- and second-order kinetics models were evaluated to fit the experimental data of the adsorption of Pb^2+^ by PEI/GO. In addition, the Weber–Morris (W-M) diffusion model was used to determine the adsorption limiting/controlling (slow) step. These models are presented in Table 2.

The adsorption capacity can be obtained using the following equation:

(2) $$q=\frac{\left(C_{i}-{\;C}_{t}\right)\times V}{m}$$

where C_i_ and C_t_ are the Pb^2+^ concentrations (mg/L) at times t = 0 and t = t, respectively. However, V and m stand for the solution volume (L) and mass of PEI/GO (g), respectively.

**Figure 8:**
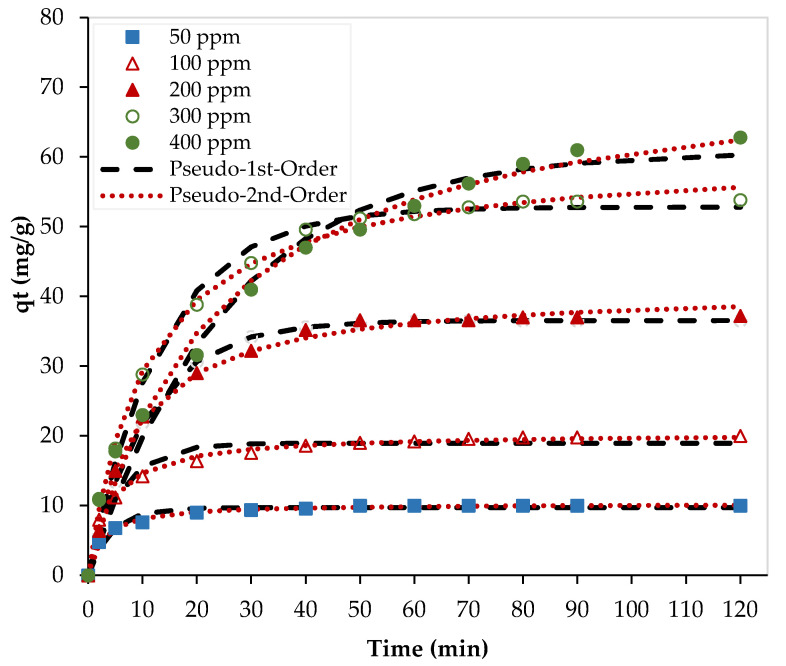
Nonlinear kinetic models. (pH = 6, PEI/GO dosage = 0.1 g, and 23 °C).

Figure 8 shows the nonlinear fittings of the experimental data at 23 °C using two kinetic models. As shown in this figure, both models (pseudo-first order and pseudo-second order) fitted well with the experimental data and kinetic parameters are presented in Table 3.

**Figure 9:**
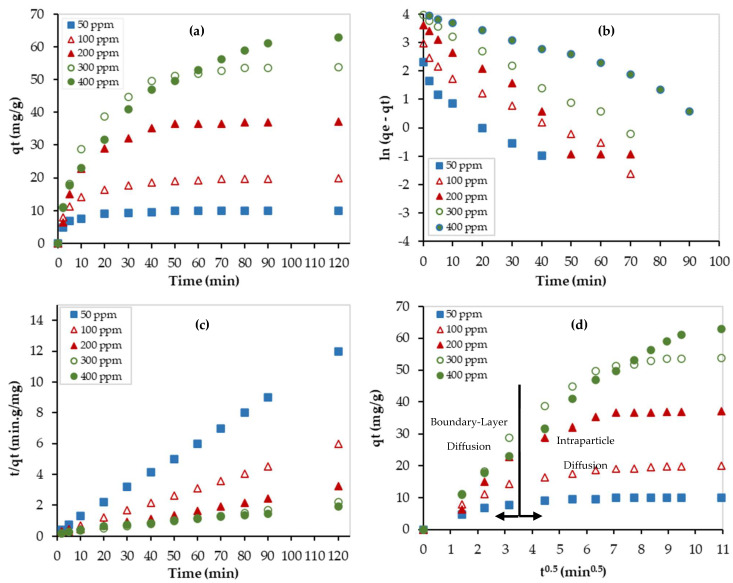
Kinetic studies for the Pb^2+^ removal using PEI/GO: (**a**) experimental data, (**b**) linear pseudo-1st-order model, (**c**) linear pseudo-2nd-order model, and (**d**) Weber–Morris model. (pH = 6, PEI/GO dosage = 0.1 g, and 23 °C).

In addition, the linear forms of the pseudo-first- and second-order kinetic models, presented in Table 3, were used to fit the experimental data at different initial Pb^2+^ concentrations (see Figure 9). Although the values of the determination coefficient (R^2^) for the pseudo-first-order model were ranging between 0.9391 and 0.9951, they were ranging between 0.9886 and 0.9997 for the pseudo-second-order model. Furthermore, the calculated values of the adsorption capacity by the pseudo-second-order model were closer to the experimental values at low adsorbate concentrations, and the obtained values by the pseudo-first order were closer to the experimental values at high adsorbate concentrations. At low Pb^2+^ concentrations (50 and 100 ppm), chemical adsorption, involving valance forces between the adsorbate and the adsorbent, was dominating. However, at high Pb^2+^ concentrations (300 and 400 ppm), physical adsorption with van der Waals forces dominates. 

Moreover, the experimental data were fitted by the W-M diffusion model, as shown in Figure 9d. Before reaching equilibrium, the data can be represented by two linear regions. While the first one represents the boundary layer diffusion zone, the second one represents the intraparticle diffusion zone. The obtained parameters of the W-M model are listed in Table 3. The R^2^ values of the second region ranged between 0.9483 and 0.9963. The effect of the diffusion boundary layer can be described based on the intercept (C) value of the M-W model. The larger the value of C, the greater the effect of the boundary layer. As presented in Table 3, the C values are large, thus indicating that the diffusion rate is controlled by the boundary layer diffusion step. In addition, the values of C almost increased as the Pb^2+^ initial concentration increased, which implies that the boundary layer diffusion step became more dominant at higher concentrations. 

**Figure 10:**
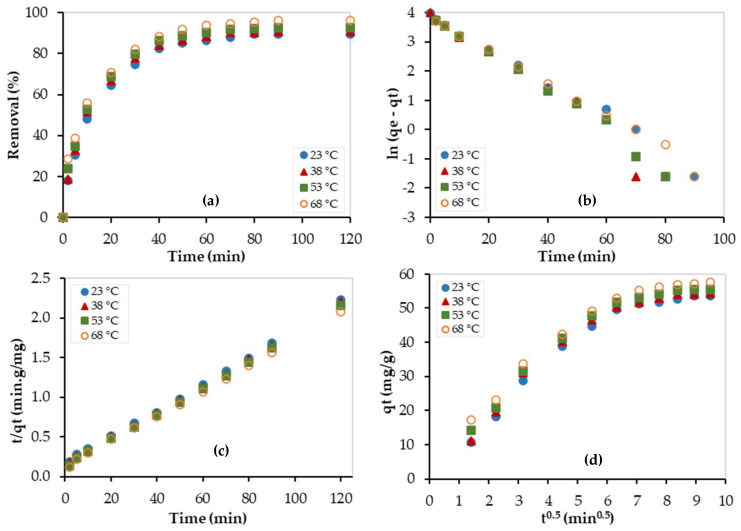
Kinetic studies for the Pb^2+^ removal using PEI/GO at different temperatures: (**a**) adsorption capacity, (**b**) pseudo-1st-order model, (**c**) pseudo-2nd-order model, and (**d**) Weber–Morris model. (pH = 6, PEI/GO dosage = 0.1 g, Pb^2+^ concentration = 300 ppm).

The kinetic study was also performed at 23, 38, 53, and 68 °C, and experimental data were fitted by the above-mentioned kinetic models, as shown in Figure 10. The obtained kinetic parameters are summarized in Table 3. As shown in Figure 10a, the adsorption capacity increased as the temperature increased, and this indicates that the adsorption process is endothermic. In addition, as temperature increased, the experimental data were best fitted with the pseudo-second-order model (see Figure 10b,c) and this implies that the chemisorption process became more dominating at high temperatures. Similarly, as shown in Table 4, the C values are large and thus indicate that the boundary layer diffusion step is the limiting step of the adsorption process. In addition, the values of C almost increased as temperature increased, indicating that the boundary layer diffusion step became more dominant at higher temperatures.

#### 3.3.2. Isotherm Study

To get more details about the adsorption process, the isotherm study is performed. For this purpose, several isotherm models are used. In this work, three of the most widely used models are used. Their equations and a brief description of their use are presented in Table 5.

In addition, the Langmuir constant (K_L_) can be used to obtain a dimensionless parameter (R_L_) that is used to determine whether the adsorption process is favorable or not.

(3) $$R_{L}=\frac{1}{1+K_{L}C_{i}}$$

**Figure 11:**
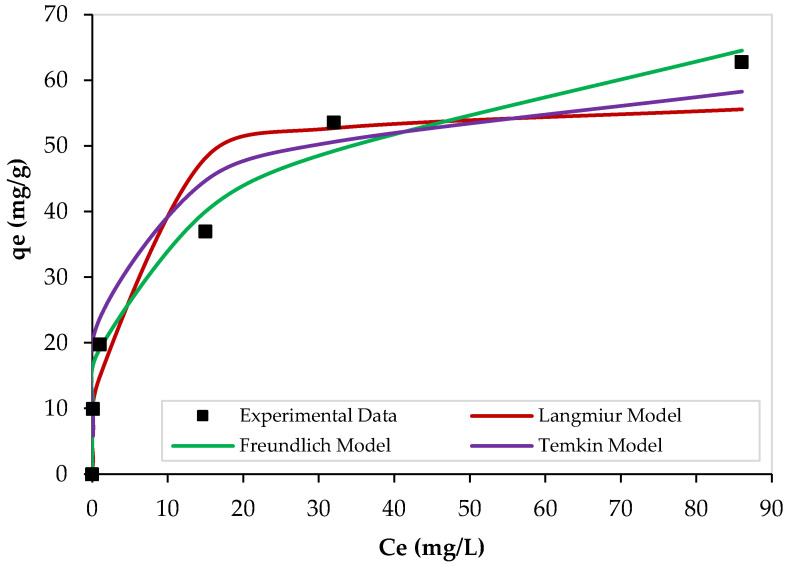
Nonlinear isotherms of Pb^2+^ adsorption over PEI/GO. (pH = 6, PEI/GO dosage = 0.1 g, and 23 °C).

The adsorption experimental data were fitted by Langmuir, Freundlich, and Temkin equations to find the most suitable isotherm model that can well-describe the process. Figure 11 illustrates the nonlinear adsorption isotherms of Pb^2+^ over PEI/GO using 0.1 g of PEI/GO at 23 °C, pH = 6, and a contact time of 120 min. At low Pb^2+^ initial concentrations, a sharp increase in the equilibrium adsorption capacity (q_e_) was noticed, and this means that the available adsorption sites on the PEI/GO surface are sufficient for Pb^2+^ ions. However, as the Pb^2+^ initial concentration increased, the change in the q_e_ values decreased. All models showed similar trends. However, the equilibrium adsorption data were better fitted by the Freundlich isotherm model when compared with other models. The parameters of the isotherm models are summarized in Table 6. 

**Figure 12:**
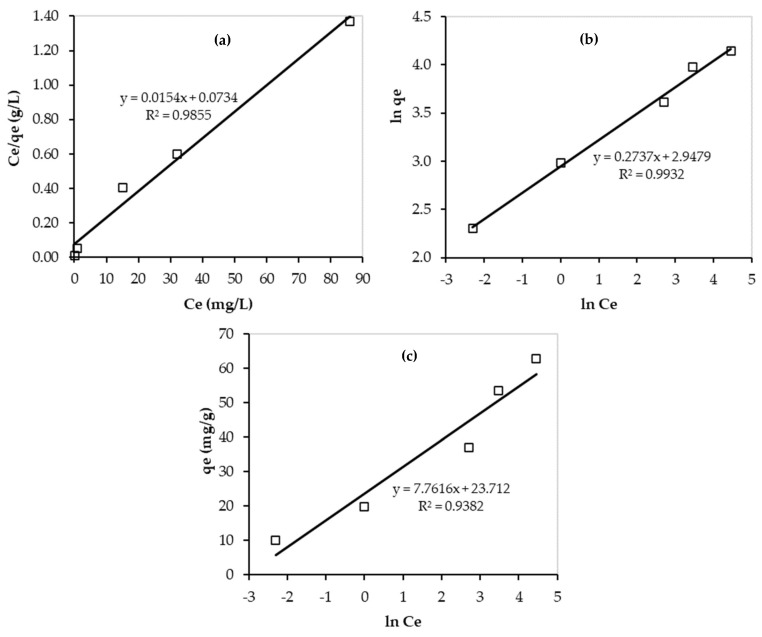
Linear isotherms of Pb^2+^ adsorption over PEI/GO: (**a**) Langmuir; (**b**) Freundlich; and (**c**) Temkin models. (pH = 6, PEI/GO dosage = 0.1 g, and 23 °C).

In addition, the experimental data were fitted by the linear forms of the isotherm models and the results are presented in Figure 12. As shown in this figure, the highest value of R^2^ was obtained for the Freundlich isotherm model (R^2^ = 0.9932). Therefore, the Pb^2+^ adsorption onto PEI/GO forms multilayers and is favorable on heterogeneous surfaces of PEI/GO, and the adsorption rate increases as adsorbate concentration increases till reaching equilibrium. The n value (3.65) confirmed the great adsorbent (PEI/GO) affinity for Pb^2+^ ions.

Furthermore, the obtained value of R_L_ is less than one but greater than zero, and thus, the adsorption process is favorable [37]. However, the very low value of R_L_ (0.07) indicates the existence of both physical and chemical adsorption of Pb^2+^ on PEI/GO during the adsorption process. This finding is supported by the kinetic results, as mentioned earlier. Moreover, the maximum adsorption capacity of Pb^2+^ on PEI/GO was 64.94 mg Pb^2+^/g PEI/GO. These good findings can result in very promising applications of PEI/GO in water and wastewater treatments. In addition, the value of b_T_ can tell about the interaction between the adsorbent and adsorbate. For the Pb^2+^ adsorption over PEI/GO, the obtained value of b_T_ is quite high (21.22 kJ/mol), which indicates a strong interaction between Pb^2+^ ions and PEI/GO.

### 3.4. Adsorption Thermodynamics 

**Figure 13:**
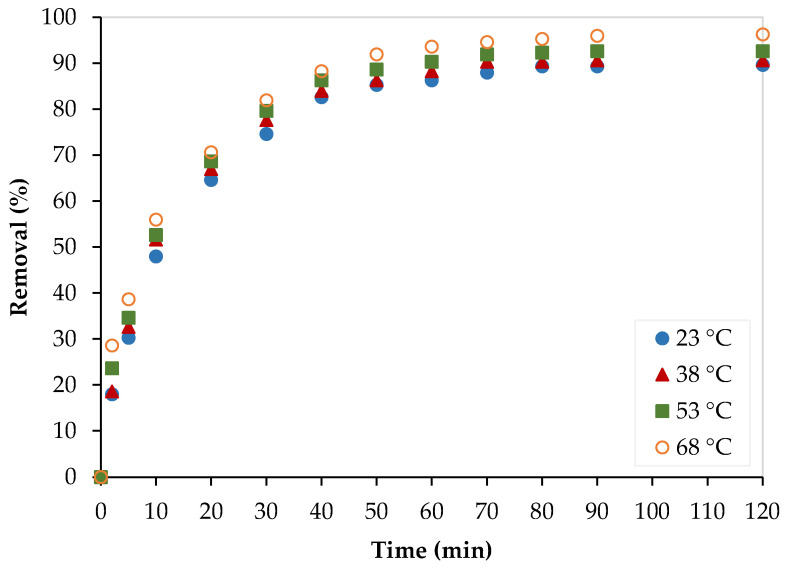
Temperature effect on the Pb^2+^ removal by PEI/GO. (pH = 6, PEI/GO dosage = 0.1 g, and Pb^2+^ initial concentration = 300 ppm).

The temperature dependence of the Pb^2+^ adsorption process over PEI/GO was assessed at four different temperatures (23, 38, 53, and 68 °C), as shown in Figure 13. The Pb^2+^ removal increased as the temperature increased, which implies the endothermic nature of the process.

In addition, more adsorption features were investigated thermodynamically at different temperatures. Specifically, the values of standard enthalpy change (ΔH°), entropy change (ΔS°), and Gibbs’s free energy change (ΔG°) during the adsorption process were targeted to be obtained using the following equations:

(4) $$K_{D}=\frac{q_{e}}{C_{e}}$$

(5) $${\ln\;K}_{D}=\frac{\Delta S^{o}}{R}-\frac{\Delta H^{o}}{R\;T}$$

(6) $$\Delta G^{o}=\Delta H^{o}-\;T\;\Delta S^{o}$$

The equilibrium constant (K_D_) was initially obtained at different temperatures using Equation (4) and then the values of ΔH°, ΔS°, and ΔG° were calculated using Equations (5) and (6). Results are shown in Table 7.

A positive value of ΔH° was reported, and this finding confirmed the endothermic nature of the adsorption process. In addition, negative values of ΔG° and a positive value of ΔS° imply the spontaneity of the adsorption process of Pb^2+^ on the PEI/GO adsorbent with a slight increase in randomness (positive ΔS°). Furthermore, the ΔG° values decreased as temperature increases, and thus, the process is energetically favorable at high temperatures.

### 3.5. Proposed Adsorption Mechanism

**Figure 14:**
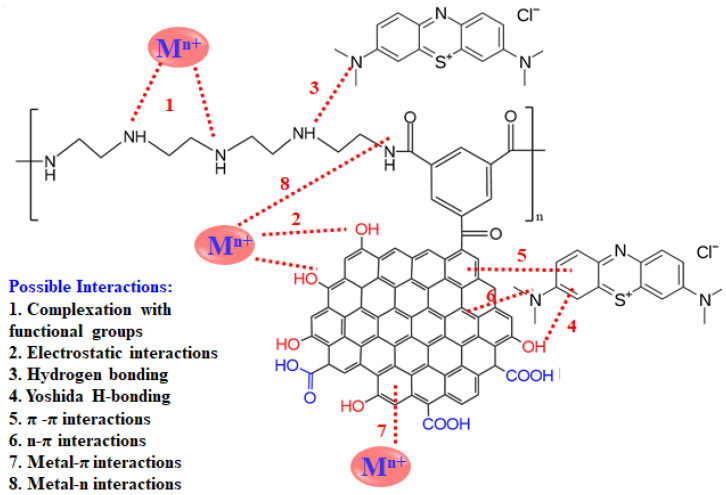
Illustration of possible mechanisms of pollutants–adsorbent interactions for the adsorption of metal ions and organic dye. (Note: some of the repeated or predefined interactions were for simplicity omitted, and M refers to metal ions).

The high efficiency of the reported adsorbent can be explained by the possible proposed mechanisms, as shown in Figure 14. The adsorption of metal ions including lead ions (Pb^2+^) and/or organic pollutants on PEI/GO involves one or more types of possible interactions. Such interactions include complexation with functional groups, electrostatic interactions, hydrogen bonding, Yoshida h-bonding, π-π interactions, n-π interactions, metal-π interactions, and/or metal-n interactions [38,39]. For instance, the lead ions are positively charged and get attracted to the negatively charged surface of the adsorbent. Functional groups on the adsorbent such as oxygen-containing groups and amines, in addition to the electron cloud of all the graphene rings, contribute significantly to the adsorption of the pollutants.

### 3.6. Comparison with Literature

The maximum adsorption capacities (q_m_) of Pb^2+^ by PEI/GO and some other adsorbents are summarized in Table 8. Compared to other reported adsorbents (commercial activated carbons [40,41,42,43,44], waste-based adsorbents/biosorbents [8,9,10,11,12,13,45,46], clays [47,48,49], magnetic nanoparticles [50], CNT composites [51], and silica-based sorbents [52,53], PEI/GO showed an excellent Pb^2+^ adsorption capacity, and this can be attributed to the distinguished features of PEI/GO and, thus, the variety of possible adsorption mechanisms (eight possible interactions between Pb^2+^ and PEI/GO), as mentioned earlier (Figure 14). 

## 4. Conclusions

In this work, a novel polyethyleneimine-grafted graphene oxide (PEI/GO) adsorbent was successfully synthesized using graphene, polyethyleneimine, and trimesoyl chloride. PEI/GO was then evaluated for the removal of Pb^2+^ from contaminated water solutions, and the following conclusions can be drawn:

- PEI/GO showed better adsorption performance when compared to graphene oxide because of the formation of polymeric branches on the graphene oxide surface and, thus, the more adsorption active sites and possible interactions with Pb^2+^ ions.
- Batch adsorption experiments confirmed that the removal % of Pb^2+^ by PEI/GO is directly proportional to the solution pH (till pH = 6), PEI/GO dosage, and contact time. However, it is inversely proportional to the Pb^2+^ initial concentrations. However, all parameters were optimized to be used for the kinetic, isotherm, and thermodynamic investigations.
- The kinetic study revealed that while chemosorption was dominating at low Pb^2+^ concentrations, physisorption was dominating at high concentrations, and the adsorption rate was controlled by the boundary-layer diffusion step.
- The adsorption process was well-described by the Freundlich isotherm model, which implies the heterogeneity of the adsorption process.
- Thermodynamic results confirmed the endothermic nature and spontaneity of the adsorption process of Pb^2+^ on PEI/GO.
- Results confirmed that PEI/GO is a highly effective adsorbent for Pb^2+^ removal from water solutions with a maximum adsorption capacity of 64.94 mg Pb^2+^/g PEI/GO, which is better than many different reported adsorbents.
- These promising results will lead to new applications of PEI/GO in water and wastewater treatments. However, more studies are recommended to investigate the reusability and stability of PEI/GO under real industrial conditions.

## Figures and Tables

**Table 1:** Advantages and disadvantages of some conventional treatment techniques used to remove water pollutants [2].

Technique	Advantages	Disadvantages
Precipitation	Simple Used for a high load of pollutants	Not selective Inefficient to remove low concentrations of metal ions High consumption of chemicals High waste (sludge) production
Ion exchange	Simple Easy to use Rapid and efficient process Effluent with excellent quality	Economical constraints Requires large columns for large volumes Rapid saturation Requires regeneration Beads fouling
Filtration/reverse osmosis	Availability of different membranes Effluent with excellent quality Rapid and efficient process	High investment cost High energy requirements Requires frequent maintenance Rapid clogging of membranes Low throughput
Coagulation-flocculation	Simple Inexpensive	Non-reusability of the used chemical High waste (sludge) production
Oxidation	Simple, rapid, and efficient Pretreatment indispensable High throughput	Requires chemicals Oxidant-dependent efficiency Short half-life Formation of undesirable intermediates Sludge generation
Adsorption	Simple Used for a wide range of water pollutants Highly efficient Selective Effluent with excellent quality Adsorbent separation and reusability Low cost	Adsorbent-dependent efficiency Requires regeneration of the used adsorbent

**Table 2:** Kinetic models.

Models	Equations	Description
Pseudo-1st-order model	$q_{t}={\;q}_{e\;}\left(1-e^{-k_{1}\;t}\right)$	Considers physisorption as the rate-limiting mechanism of the adsorption process [31].
Pseudo-2nd-order model	$q_{t}=\frac{q_{e}^{2}{\;k}_{2}\;t}{1+{\;q}_{e\;}{\;k}_{2}\;t}$	Considers chemisorption as the rate-limiting mechanism of the adsorption process [32].
Weber–Morris (W-M) diffusion model	$q_{t}=K_{\mathrm{id}}{\;t}^{1/2}+C$	Considers intraparticle diffusion as the rate-limiting mechanism of the adsorption process [33].

Where q_t_, and q_e_ are the adsorption capacities of Pb^2+^ over PEI/GO (mg Pb/g PEI/GO) at the time (t) and equilibrium, respectively.

**Table 3:** Kinetic parameters for Pb^2+^ adsorption over PEI/GO (pH = 6, PEI/GO dosage = 0.1 g, and 23 °C).

Models	Linear Equations	Parameters	Values				
			Pb^2+^ Initial Concentration (mg/L)				
			50	100	200	300	400
Pseudo-1st-order model	$\ln\left(q_{e}-q_{t}\right)=\ln q_{e}-k_{1}t$	q_e_, exp (mg/g)	9.98	19.8	37	53.6	62.8
		k_1_ (min^−1^)	0.246	0.174	0.091	0.074	0.040
		q_e_, cal (mg/g)	9.70	18.94	36.53	52.80	60.81
		R^2^	0.9391	0.9751	0.9625	0.9951	0.9748
Pseudo-2nd-order model	$\frac{t}{q_{t}}=\frac{1}{k_{2}{\;q}_{e}^{2}}+\frac{t}{q_{e}}$	k_2_ (g/mg·min)	0.038	0.012	0.003	0.002	0.001
		q_e_, cal (mg/g)	10.27	20.42	41.23	60.58	74.26
		R^2^	0.9997	0.9997	0.9981	0.9986	0.9886
W-M diffusion model	$q_{t}=K_{\mathrm{id}}{\;t}^{1/2}+C$	K_id_ (mg/g·min^1/2^)	0.3195	0.8755	3.0012	1.4736	4.594
		C (mg/g)	7.6095	12.716	15.734	40.467	17.594
		R^2^	0.9666	0.9483	0.9887	0.985	0.9963

**Table 4:** Kinetic parameters of the adsorption of Pb^2+^ over PEI/GO. (pH = 6, PEI/GO dosage = 0.1 g, Pb^2+^ concentration = 300 ppm).

Models	Parameters	Values			
		Temperature (°C)			
		23	38	53	68
Pseudo-1st-order	q_e_, exp (mg/g)	53.8	54.4	55.6	57.8
	k_1_ (min^−1^)	0.0615	0.0694	0.0665	0.0577
	q_e_, cal (mg/g)	52.05	55.12	52.79	48.68
	R^2^	0.9822	0.9731	0.9905	0.9941
Pseudo-2nd-order	k_2_ (g/mg·min)	0.0018	0.0020	0.0021	0.0022
	q_e_, cal (mg/g)	59.17	59.52	60.24	62.11
	R^2^	0.9986	0.9987	0.9987	0.9989
Weber–Morris (W-M) diffusion model	K_id_ (mg/g·min^1/2^)	1.3098	1.8514	1.5429	1.0639
	C (mg/g)	9.2809	38.692	42.277	47.805
	R^2^	0.9865	0.9994	0.9994	0.972

**Table 5:** Isotherm models.

Models	Equations	Description
Langmuir	$q_{e}=\frac{{\;q}_{m\;}K_{L\;}{\;C}_{e}}{1+K_{L\;}C_{e}}$	Evaluates the process on a homogeneous monolayer without interaction between adsorbed ions [34]
Freundlich	$q_{e}={\;K}_{F\;}C_{e}{}^{\frac{1}{n}}$	Evaluates heterogeneous multilayer adsorption with the interaction between adsorbed ions [35]
Temkin	$q_{e}=\frac{\mathrm{RT}}{b_{T}}\ln\;(K_{T}{\;C}_{e})$	Evaluates the interaction between adsorbent and adsorbate [36]

Where q_m_ is the maximum adsorption capacity (mg Pb^2+^/g PEI/GO), K_L_ is a constant of the Langmuir model (L/mg), C_e_ is the Pb^2+^ equilibrium concentration (mg/L), K_F_ and n are the Freundlich model constants, R is the gas constant, and b_T_ and K_T_ are the Temkin isotherm constants.

**Table 6:** Isotherm parameters of the Pb^2+^ adsorption on PEI/GO. (pH = 6, PEI/GO dosage = 0.1 g, and 23 °C).

Models	Linear Equations	Parameters	Values	R^2^
Langmuir	$\frac{C_{e}}{q_{e}}=\frac{1}{K_{L\;}{\;q}_{m}}+\frac{C_{e}}{q_{m}}$	q_m_ (mg/g)	64.94	0.9855
		K_L_ (L/mg)	0.21	
		R_L_	0.04	
Freundlich	${\ln\;q}_{e}={\ln\;K}_{F}+\frac{1}{n}{\ln\;C}_{e}$	n	3.65	0.9932
		K_F_ (mg/g)	19.07	
Temkin	$q_{e}=\frac{\mathrm{RT}}{b_{T}}{\ln\;K}_{T}+\frac{\mathrm{RT}}{b_{T}}{\ln\;C}_{e}$	K_T_ (L/g)	0.32	0.9382
		b_T_ (kJ/mol)	21.22	

**Table 7:** Thermodynamic properties of the Pb^2+^ adsorption on PEI/GO. (pH = 6, PEI/GO dosage = 0.1 g, and Pb^2+^ initial concentration = 300 ppm).

Property	Temperature (°C)			
	23	38	53	68
ΔG° (kJ/mol)	−1.00	−2.05	−3.10	−4.15
ΔH° (kJ/mol)	19.73			
ΔS° (J/mol·K)	0.07			

**Table 8:** The maximum adsorption capacity of Pb^2+^ by various adsorbents.

Adsorbent	q_m_ (mg/g)	Reference
Commercial activated carbon (AC)	54.65	Krishnan et al. (2003) [40]
AC	35	Xu and Liu (2008) [41]
AC	43.86	Acharya et al. (2009) [42]
AC	27.53	Momcilovic et al. (2011) [43]
AC nanocomposite	14	Fernando et al. (2015) [44]
Palm kernel fiber	40.20	Ho and Ofomaja (2005) [45]
Algal waste	44	Vilar et al. (2005) [46]
Albizia lebbeck pods	7.17	Mustapha, et al. (2019) [8]
Cassava peels	50.1	Thompson et al. (2020) [9]
Brewed tea waste	1.2	Çelebi et al. (2020) [10]
Sugarcane bagasse	1.61	Ezeonuegbu, et al. (2021) [11]
Mango seeds cover with kernel Jamun seeds cover with kernel	39.15 20.28	Pall et al. (2022) [12]
Parkia speciosa pod	48.7	Tee et al. (2022) [13]
Turkish kaolinite clay	31.75	Sari et al. (2007) [47]
Natural bentonite	32.68	Melichová, Z.; Hromada, L. (2013) [48]
Bentonite composite	4.6	Mo et al. (2017) [49]
Montmorillonite coated by amino magnetic nanoparticles	38.15	Irawan et al. (2019) [50]
CNTs–PAMAM–Ag	18.7	Neelgund et al. (2022) [51]
Silica-coated magnetic nanocomposites	14.9	Nicola et al. (2020) [52]
Ordered mesoporous silica	18.8	Putz et al. (2022) [53]
Polyethyleneimine -grafted graphene oxide	64.94	This work

## Data Availability

Not applicable.
